# Experiencing COVID-19 at a large district level hospital in Cape Town: A retrospective analysis of the first wave

**DOI:** 10.4102/sajid.v37i1.317

**Published:** 2022-03-29

**Authors:** Nadè Claassen, Gerhard van Wyk, Sanet van Staden, Michiel M.D. Basson

**Affiliations:** 1 Department of Internal Medicine, Karl Bremer Hospital, Cape Town, South Africa

**Keywords:** COVID-19, mortality, comorbidities, admissions, first wave of infections, district level hospital, diabetes, hypertension

## Abstract

**Background:**

The coronavirus disease 2019 (COVID-19) pandemic in tertiary hospitals from South Africa and world wide have been well described, but limited data are published on the findings. This article aimed to describe patients admitted to a large district hospital in Cape Town, South Africa, during the first wave of severe acute respiratory syndrome coronavirus 2 (SARS-CoV-2) Infections. To compare the clinical features and further investigate survivors and deceased COVID-19 patients.

**Methods:**

A single centre retrospective review of clinical records and laboratory data of patients admitted with a positive SARS-CoV-2 polymerase chain reaction (PCR) from April 2020 to August 2020.

**Results:**

A total of 568 patients with a positive SARS-CoV-2 PCR were admitted to the study centre for one night or longer and of these patients 154 (27%) died of COVID-19. The median age of patients who died of COVID-19 was 66 years and 53 years for survivors. Hypertension, diabetes mellitus and obesity were the commonest comorbidities in patients who survived and died of COVID-19. There were no major differences when comparing the severity of infiltrates on chest X-rays (CXR) of COVID-19 survivors with deceased patients. More than half (58%) of deceased patients died within 3 days following admission to hospital. A substantial number of patients who died of COVID-19 had associated acute kidney injury (*n* = 79, 51%).

**Conclusion:**

Acute kidney injury had a high prevalence amongst patients who died of COVID-19. Delays in transfer to intensive care unit (ICU), limited ICU capacity and disease severity contributed to a substantial number of patients dying within 3 days of admission.

## Introduction

Since the advent of the outbreak of severe acute respiratory syndrome coronavirus 2 (SARS-CoV-2) worldwide, the lives of more than five million individuals have been lost because of coronavirus disease 2019 (COVID-19).^1^ Worldwide the case fatality rate is estimated to be 2.0% – 3.4%.^1^ Models estimate the South African case fatality rate to be comparable with the rate reported in the rest of the world. The under-reporting of cases and deaths, limited availability of testing in rural areas and different testing strategies are challenges impeding the calculation of a more accurate case fatality rate. When looking at the South African Medical Research Council data on excess deaths since the start of the pandemic, the official South African death rate seems to be significantly underestimated.^2,3^

The South African Healthcare System poses unique challenges in terms of limited fiscal resources and infrastructure, stark disparity between public and private health sectors, understaffing and the so-called quadruple burden of disease (namely, maternal and child deaths, human immunodeficiency virus (HIV) or acquired immunodeficiency syndrome (AIDS) and tuberculosis [TB], non-communicable diseases and trauma and injury).^4,5^ Poverty, illiteracy and inequality account for even more strain on the healthcare system, leading to poor access to healthcare, overcrowding and ultimately the spread of SARS-CoV-2 virus amongst individuals from vulnerable communities.^6^ Furthermore, South Africa has seen an increase in obesity and diseases of lifestyle amongst low-income classes over recent years.^7^

The COVID-19 pandemic has brought the spotlight on sub-Saharan Africa and what the response will be in a population already under the heavy burden of HIV and AIDS, TB, and non-communicable diseases. With primary and district healthcare services having even more limited resources and bed capacity,^8,9,10,11^ mechanisms had to be put in place to ensure the appropriate management and referral of patients with COVID-19 who are critically ill. This also raised concern for primary care platforms servicing people living with HIV, TB and non-communicable diseases being side-tracked by an overwhelming number of COVID-19 cases.

Much has been learned in terms of disease process and therapeutics since the first wave and several factors associated with severe COVID-19 have been identified. Amongst these factors are demographic factors (male gender, smoking status and increasing age), biochemical and radiological parameters and the sequential organ failure assessment (SOFA) score.^12,13,14,15,16,17,18^ Certain comorbidities are also associated with increased risk of severe COVID-19 and risk of death,^12,13,14,15,16,17^ with knowledge regarding the interplay between diseases such as HIV and AIDS and associated opportunistic infections and COVID-19 growing by the day.^15,17,19,20,21,22,23^

The Western Cape and specifically the Cape Town metropole was the area hit the hardest in the initial stages of the pandemic in South Africa, its peak being towards the end of June 2020.^24^ The province’s COVID-19 response was met with several challenges and triumphs, but despite a robust contingency plan in place, the lives of thousands of patients were lost to COVID-19.^20^

The subsequent second and third waves of SARS-CoV-2 infections were predominated by the new and more transmissible Beta (B.1.351) and Delta (B.1.617.2) variants, which are also known to cause more severe disease in infected individuals.^25,26,27^ With a fourth wave of COVID-19 infections looming, only a small fraction of the South African population vaccinated against COVID-19 and new SARS-CoV-2 variants arising rapidly, it is uncertain for how long the pandemic will continue to cause death and devastation around the world.^28,29^ We compare a cohort of patients who died of COVID-19 (either because of direct effects or consequences of disease) with COVID-19 survivors in terms of clinical, radiological and biochemical parameters.

## Methods

### Study design and setting

This was a single centre study that included 573 admissions to a large district level hospital in Cape Town, South Africa, during the so-called first wave of SARS-CoV-2 infections. The study hospital services the Northern as well as a part of the Tygerberg substructures of Cape Town, which has a total adult population of 870 826. However, a part of the Tygerberg Substructure drains directly to the Tygerberg Academic Hospital (TBH) and an unknown portion of the population used services provided by the private healthcare sector. The true population size reflected in this cohort is therefore uncertain.

The study site is a district level hospital with 282 beds and a 4-bed high care unit (HCU). The COVID-19 cases admitted to the hospital were primarily managed by the hospital’s department of internal medicine. This department was staffed with 2 full time specialist physicians and 11 medical officers and a registrar in internal medicine at the time of this study.

The study included all patients aged 18 years or older with a positive SARS-CoV-2 polymerase chain reaction (PCR) test who were admitted to hospital from 01 April 2020 to 31 August 2020. Severe acute respiratory syndrome coronavirus 2 antigen tests were not yet available for use during the study period. All patients admitted during the study period were included in this comparison, including the large number of patients who were transferred to intermediate care facilities (for further management once stable and acute complications requiring specialist care were ruled out or treated) as well as patients who were transferred to facilities providing a higher level of care or palliative care. Patients were separated into two groups, namely survivors (those who were discharged alive from either the base hospital or the hospital they were transferred to) and COVID-19 deaths (those patients who died during their admission to either the base or referral hospital).

Two burden of disease surveys were carried out during the year 2020 – one just before the first wave of the COVID-19 pandemic in February 2020 and one during the peak of the first wave in June 2020. Data from these surveys were used to assess and compare the number of admissions and admission diagnoses other than COVID-19.

### Data collection

Patients were identified using the hospital’s admissions registry for the duration of the study period. Inclusion criterion was all patients aged 18 years or older with a positive SARS-CoV-2 PCR admitted to the hospital for one night or longer regardless of the reason for admission. Patient outcomes were checked to determine the number of deaths, discharges and transfers to other facilities using the admission lists, the electronic continuity of care record (eCCR) and Clinicom patient administration system. All patients who were discharged alive (whether from the study centre or transfer hospital) were included in the survivor analysis group and all patients who died of COVID-19 during their admission (whether at the study centre or at hospitals elsewhere after being transferred) were included in the COVID-19 death analysis group.

Data were extracted from patient files and laboratory records, using a standardised form, including demographic details (age, gender and race), comorbidities (hypertension, diabetes mellitus, HIV infection, active TB, chronic lung disease, chronic kidney disease, cardiovascular disease, dyslipidaemia, connective tissue disease, chronic neuromuscular disease, active malignancy, smoking, obesity, etc.), timing of onset of symptoms to admission and death, clinical features on admission (pulse oximetry, whilst breathing ambient air where available), blood results (full blood and differential counts, serum creatinine, C-reactive protein), complications (acute kidney injury [AKI], acute arterial occlusion, congestive cardiac failure, diabetic ketoacidosis (DKA), haematological abnormalities, healthcare associated infection, hypernatraemia and dehydration, hypoglycaemia and venous thromboembolism) and whether or not corticosteroids were administered.

Blood results were interpreted according to National Health Laboratory Services reference values.

Comorbidities were further assessed to get an indication of disease control. Patients with diabetes mellitus were assessed by means of glycated haemoglobin (HbA1c) carried out during admission or within four months prior to admission. Target organ damage (if present) was specifically mentioned. Pharmacy records were audited to assess how many antihypertensive agents patients were on. Patients with HIV-infection were assessed in terms of their most recent CD4+ lymphocyte count, whether they were on antiretroviral medication and their viral loads. Patients were classified as obese or not according to the admitting physician’s clinical impression. Acute kidney injury was defined according to the Kidney Disease Improving Global Outcomes (KDIGO) Clinical practice guidelines’ definition of AKI.^30^

Data from two of the Department of Internal Medicine’s burden of disease surveys during the months of February 2020 and June 2020 were included to assess and compare admission diagnoses and patient numbers. This survey is an audit of all inpatients admitted to the internal medicine service. Patients were identified using the hospital’s admissions registry that provided information on primary and secondary diagnoses, patient outcomes and length of stay.

Due to the nature of the study, consent was not obtained. Patient confidentiality was ensured by means of anonymising data.

### Statistical analyses

Data were collected and analysed using Microsoft Excel. Normally distributed descriptive numerical data were described using means and standard deviations (s.d.) and non-normally distributed data were described using medians and interquartile ranges (IQRs). Continuous data were compared using the *t*-test for data with a normal distribution and the Mann–Whitney U test for data with a non-normal distribution. Odds ratios (ORs) (unadjusted) were calculated using bivariate analysis to assess association between exposure and outcome. Statistical significance was set at *p* < 0.05 and confidence interval of 95% was used.

### Ethical considerations

The study was submitted and approved by the South African Medical Association Research and Ethical Committee (SAMAREC). The SAMAREC is associated with the South African Medical Association.

## Results

**FIGURE 1:**
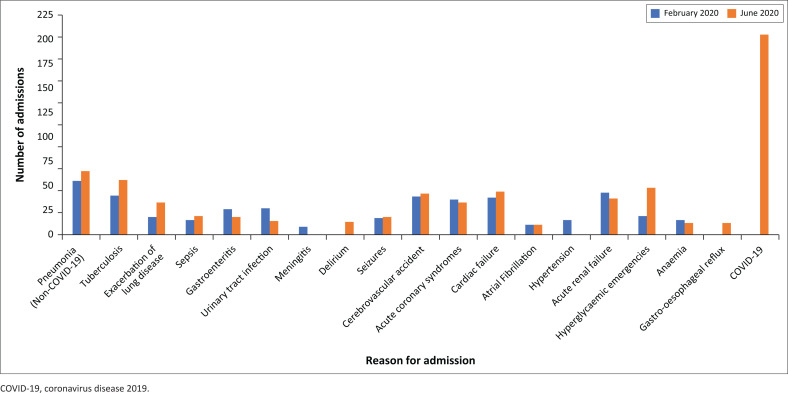
Comparison of reasons for admission to internal medicine during the months of February 2020 and June 2020.

The department’s two burden of disease surveys during February 2020 and June 2020 were compared to assess the different reasons for admission to the study centre’s internal medicine service (Figure 1). From this comparison it is evident that the COVID-19 cases admitted to the study centre were additional to the usual number of admissions, as there was little difference in the number of admissions to internal medicine for diagnoses other than COVID-19 between the months of February 2020 and June 2020.

**FIGURE 2:**
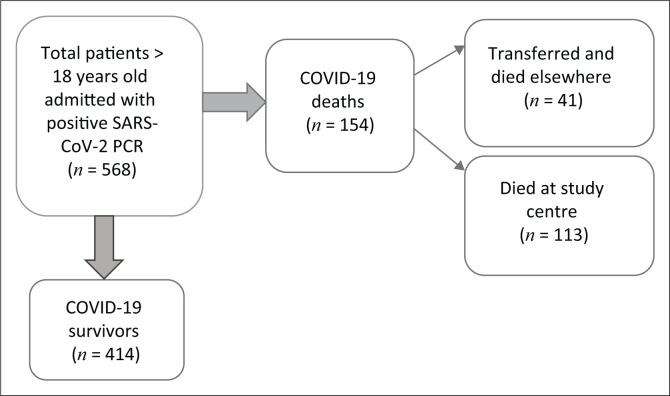
Flow diagram detailing number of patients admitted, survived and died.

A total of 568 patients with laboratory confirmed COVID-19 were admitted to the hospital’s COVID-19 service for one night or longer from 01 April 2020 to 31 August 2020. All were included in this study. A total of 154 patients died whilst admitted to the study centre or after being transferred to other facilities because of direct effects or complications of COVID-19, equating to a case fatality rate of 27% for patients requiring admission. A total of 41 of the 154 patients were transferred alive to other facilities (whether for a higher level of care, intermediate or palliative care) and died there.

The median (IQR) age of patients who died of COVID-19 was 66 (IQR: 55–75) and more males (*n* = 91) died compared with females (*n* = 63). Amongst COVID-19 survivors admitted to the study centre during the study period, the median (IQR) age was 53 (IQR: 37–64).

Of the 154 patients who died the majority were mixed race (45%; *n* = 69) and black people (38%; *n* = 59), followed by white people (16%; *n* = 24) and one Asian. The race of one patient was not clear.

### Comorbidities

Amongst patients admitted to the study centre with COVID-19, the most common comorbidities included hypertension, diabetes mellitus, obesity, cardiovascular disease, smoking, chronic lung disease, chronic kidney disease and HIV-infection (Table 1). It is our impression that certain comorbidities such as obesity was under-reported as weight and height was not consistently documented on admission and due to the variability in the clinical impression and note keeping of treating doctors.

**TABLE 1:** Comparison of comorbid conditions in patients who died of COVID-19 and those who were discharged alive (survivors).

Patient characteristics	Died (*N* = 154)						Survived (*N* = 414)						OR	95% CI	*p*
	*n*	%	Median	IQR	Mean	s.d	*n*	%	Median	IQR	Mean	s.d			
**Age**	-	-	66	55–75	-	-	-	-	53	37–64	-	-	9.2–15.4	-	< 0.0001
**Gender**															
Male	91	59.0	-	-	-	-	178	43.0	-	-	-	-	1.9	1.3–2.8	0.0007
Female	63	41.0	-	-	-	-	236	57.0	-	-	-	-	0.5	0.4–0.8	0.0007
**Race**															
Asian people	1	0.6	-	-	-	-	N/A	-	-	-	-	-	N/A	-	N/A
Black people	59	38.3	-	-	-	-	N/A	-	-	-	-	-	N/A	-	N/A
Mixed race people	69	44.8	-	-	-	-	N/A	-	-	-	-	-	N/A	-	N/A
White people	24	15.6	-	-	-	-	N/A	-	-	-	-	-	N/A	-	N/A
Unknown	1	0.6	-	-	-	-	N/A	-	-	-	-	-	N/A	-	N/A
**Number of comorbidit ies**															
None	4	3.0	-	-	-	-	137	33.1	-	-	-	-	0.05	0.02–0.1	< 0.0001
At least one comorbidity	150	97.0	-	-	-	-	277	67.0	-	-	-	-	18.5	6.7–51.1	< 0.0001
Two or more comorbidities	130	84.0	-	-	-	-	194	47.0	-	-	-	-	6.1	3.8–9.9	< 0.0001
Three or more comorbidities	95	62.0	-	-	-	-	114	28.0	-	-	-	-	4.2	2.9–6.3	< 0.0001
**Specific comorbidities**															
Hypertension	102	66.0	-	-	-	-	157	38.0	-	-	-	-	3.2	2.2–4.7	< 0.0001
Diabetes mellitus	79	51.0	-	-	-	-	117	28.0	-	-	-	-	2.7	1.8–3.9	< 0.0001
HbA1c	-	-	-	-	9.5	2.6	N/A	-	-	-	-	-	N/A	-	N/A
Insulin dependent	26	33.0	-	-	-	-	N/A	-	-	-	-	-	N/A	-	N/A
Obesity	57	37.0	-	-	-	-	77	19.0	-	-	-	-	2.6	1.7–3.9	< 0.0001
HIV-Infection	18	12.0	-	-	-	-	46	11.0	-	-	-	-	1.1	0.5–1.9	0.85
CD4+ count	-	-	134	30–248	-	-	N/A	-	-	-	-	-	N/A	-	N/A
WHO clinical stage	-	-	-	-	3.2	0.8	N/A	-	-	-	-	-	N/A	-	N/A
On ART (% of HIV-infected)	17	94.0	-	-	-	-	N/A	-	-	-	-	-	N/A	-	N/A
**Viral load**															
VL < 1000 (% of patients on ARTs)	13	76.0	-	-	-	-	N/A	-	-	-	-	-	N/A	-	N/A
VL > 1000 (% of patients on ARTs)	4	24.0	-	-	-	-	N/A	-	-	-	-	-	N/A	-	N/A
Chronic lung disease	29	18.0	-	-	-	-	48	12.0	-	-	-	-	1.8	1.1–2.9	0.03
Chronic kidney disease	25	16.0	-	-	-	-	17	4.0	-	-	-	-	4.5	2.4–8.6	< 0.0001
Smoking	32	21.0	-	-	-	-	19	5.0	-	-	-	-	5.5	3.0–10.0	< 0.0001
Cardiovascular disease	53	34.0	-	-	-	-	39	9.0	-	-	-	-	5.1	3.2–8.1	< 0.0001
Dyslipidaemia	60	39.0	-	-	-	-	42	10.0	-	-	-	-	5.7	3.6–9.0	< 0.0001
Active malignancy	7	5.0	-	-	-	-	11	3.0	-	-	-	-	1.7	0.7–4.6	0.26
Active tuberculosis	6	4.0	-	-	-	-	11	3.0	-	-	-	-	1.5	0.5–4.1	0.44
Connective tissue disease	8	5.0	-	-	-	-	6	1.0	-	-	-	-	3.7	1.3–10.9	0.02
Neuromuscular disease	12	8.0	-	-	-	-	34	8.0	-	-	-	-	0.9	0.5–1.9	0.9

IQR, interquartile range; s.d., standard deviation; OR, odds ratio (unadjusted); CI, confidence interval, WHO, World Health Organization; ART, antiretroviral therapy; N/A, data not available; VL, viral load; HbA1c, glycated haemoglobin.

Amongst the cohort of COVID-19 deaths, 79 (51%) of the patients had either pre-existing (81%; *n* = 64) or a new diagnosis (19%; *n* = 15) of diabetes mellitus with a mean HbA1c of 9.5% (s.d.: 2.6). Of the known diabetic patients, 26 patients (33%) were insulin dependent and 25 (32%) patients had established target organ damage. A total of 16 patients presented to hospital with diabetic hyperglycaemic emergencies (DKA or hyperosmolar hyperglycaemic state [HHS]).

Amongst all patients admitted with COVID-19 the HIV seroprevalence was 11.3% (*n* = 64). Coronavirus disease 2019 survivors had an HIV seroprevalence of 11.1% (*n* = 46) and amongst patients who died of COVID-19 the seroprevalence was 11.7% (*n* = 18). Patients who died of COVID-19 had a median CD4+ lymphocyte count of 134 (*n* = 18) (IQR: 30–248) and mean World Health Organization (WHO) clinical stage of 3.2 (s.d.: 0.79). Thirteen patients were on antiretroviral therapy (ART) with suppressed viral loads. One patient was ART naive and four patients were failing their ART regimen. Two patients had a history of previous TB and four patients had active pulmonary or disseminated TB at the time of admission.

### Clinical and radiological features on admission

Patients were assessed in terms of clinical and radiological features on admission with specific mention of saturation whilst breathing ambient air and findings of plain chest X-rays (CXR). Patients who died of COVID-19 (*n* = 110) had a median saturation of 85% (IQR: 75–92) whether whilst breathing ambient air or receiving supplemental oxygen. The admission saturation levels of survivors were not reviewed in this study.

The plain CXR of patients admitted at the study centre were classified as having minimal/no infiltrates, having basal and/or peripheral infiltrates (< 50% of lung fields), diffuse bilateral infiltrates (> 50% of lung fields) or having findings not typical of COVID-19 (Table 2). Furthermore, CXRs were classified as having signs of pre-existing structural lung disease and cardiomegaly.

**TABLE 2:** Comparison of special investigations: Baseline basic laboratory results and plain CXR (describing extent of infiltrates and additional findings) in patients who survived and died of COVID-19.

Special investigations	Died (*N* = 154)					Survived (*N* = 414)					*p*
	*n*	%	Median	IQR	95% CI	*n*	%	Median	IQR	95% CI	
**Basic laboratory investigations**
C-Reactive protein (mg/L)	148	-	169	97–280	147–190	346		102	43–183	89–114	< 0.0001
(Ref < 10)											
White cell count (×10⁹/L)	148	-	9.65	6.81–13.99	8.6–10.69	387		8.28	6.08–10.75	7.84–8.72	< 0.0001
(Ref 3.92–10.4)											
Neutrophil count (×10⁹/L)	138	-	7.76	5.45–11.75	6.86–8.66	345		6.35	4.3–8.96	5.91–6.79	0.0001
(Ref 1.6–6.98)											
Lymphocyte count (×10⁹/L)	138	-	0.97	0.6–1.45	0.86–1.08	344		1.13	0.82–1.54	1.06–1.2	0.016
(Ref 1.4–4.2)											
Neutrophil/lymphocyte ratio	138	-	8.45	4.62–13.4	6.29–10.6	344		5.3	3.46–9.03	4.56–6.05	< 0.0001
**Plain X-rays**											
Extent of infiltrates	-	-	-	-	-	-	-	-	-	-	-
Minimal / no infiltrates	16	10	-	-	-	131	8	-	-	-	-
Infiltrates less than 50% of lung fields	86	55	-	-	-	196	62	-	-	-	-
Infiltrates more than 50% of lung fields	39	25	-	-	-	56	21	-	-	-	-
Not typical of COVID-19	5	3	-	-	-	3	3	-	-	-	-
No CXR	8	7	-	-	-	28	7	-	-	-	-
Additional findings:		-	-	-		-	-	-	-
Structural lung disease	13	8	-	-	-	45	8	-	-	-	-
Cardiomegaly	10	6	-	-	-	14	5	-	-	-	-

%, percentage of total; IQR, interquartile range; CI, confidence interval; CXR, chest X-ray.

### Special investigations

Commonly used special investigations were recorded and compared with that of patients admitted to the study centre who survived COVID-19 during the study period.

The baseline laboratory investigations of admitted patients who survived and died of COVID-19 were compared (Table 2).

### Disease course and complications

**FIGURE 3:**
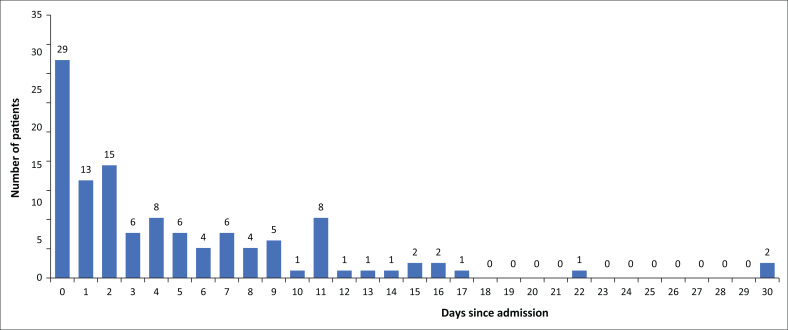
Timing of death since the day of admission. *N* = 149.

Timelines from symptom onset to testing, admission and date of death were recorded for the patients who died of COVID-19. The median (IQR) time from onset of symptoms to being tested was four days (IQR: 2–7) (*n* = 127), median (IQR) from onset of symptoms to admission five days (IQR: 2–7) (*n* = 127) and median (IQR) time from onset of symptoms until death was seven days (IQR: 5.0–12.5; *n* = 127) (Figure 3). More than 58% of the 154 patients (*n* = 90) died within 3 days of being admitted to hospital.

**FIGURE 4:**
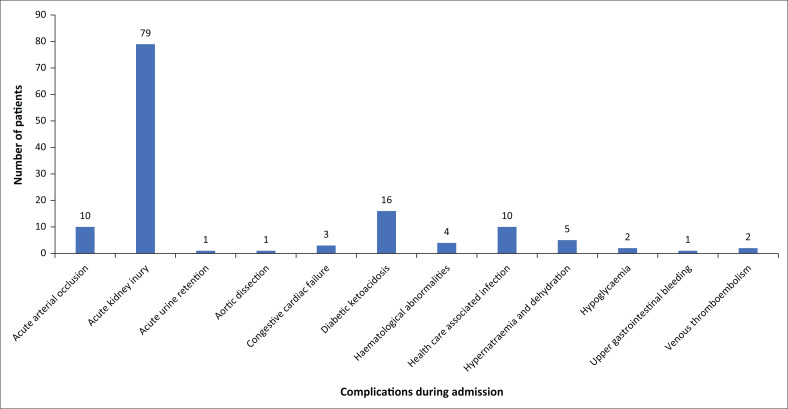
Acute complications amongst patients who died of COVID-19.

Complications whilst in hospital were identified by auditing clinical notes and laboratory data. It is our impression that complications might be under-reported as the data reflect note keeping. The most common documented complication was AKI. Laboratory data were audited to demonstrate an acute deterioration in renal function from baseline using the KDIGO definition of AKI defined by at least 1.5-fold increase in serum creatinine (SCr), increase in SCr of 26.5 µmol/L or more or urine output less than 0.5 mL/kg per hour for 6 h.^30^ A total of 79 (51%) of patients who died of COVID-19 had AKI compared with 61 (15%) of COVID-19 survivors. Amongst COVID-19 deaths with documented AKI 20 (13%) had additional complications including electrolyte disturbances and other forms of organ dysfunction. Other acute complications amongst COVID-19 deaths included healthcare associated infection (*n* = 10), DKA or HHS (*n* = 16), haematological abnormalities (such as pancytopenia and disseminated intravascular coagulation) (*n* = 4), acute arterial occlusion (*n* = 10), congestive cardiac failure (*n* = 3), hypernatremia with dehydration (*n* = 5), hypoglycaemia (*n* = 2), acute urine retention (*n* = 1) and aortic dissection (*n* = 1). Only two cases of venous thromboembolism were reported amongst the cohort of patients who died of COVID-19 (Figure 4). One patient who died of COVID-19 had a documented upper gastrointestinal haemorrhage during admission.

### Therapeutics and glucocorticoid therapy

All patients admitted to the study centre were treated according to standard of care with supplemental oxygen as required to maintain adequate oxygen saturation, antipyretics, analgesia and low molecular weight heparin in prophylactic or therapeutic doses, which was individualised on a patient-to-patient basis, unless specifically contraindicated. At the time of this study, although there was still uncertainty regarding the benefit of using thiamine and ascorbic acid most patients were treated with it. Patients were treated with broad spectrum antibiotics for community acquired pneumonia until a positive SARS-CoV-2 PCR result was obtained. Antibiotics were continued if secondary community or hospital acquired infection was suspected, where clinically indicated. A total of 51 of the 154 (33%) patients who died of COVID-19 received glucocorticoid therapy, compared with 248 (60%) of patients who survived (Table 3).

**TABLE 3:** Risk factors for death.

Risk factors for death	Survivors (*N* = 414)		Non-survivors (*N* = 154)		OR	95% CI	*p*
	*n*	%	*n*	%			
Acute renal failure	61	15	79	51	5.7	3.74–8.63	< 0.0001
Glucocorticoid administration	248	60	51	33	0.3	0.23–0.49	< 0.0001
Age ≥ 50	234	57	128	83	3.8	2.38–6.02	< 0.0001
Age ≥ 55	178	42	119	77	4.5	2.95–6.89	< 0.0001
Age ≥ 60	136	33	109	71	5.0	3.31–7.41	< 0.0001
Age ≥ 65	95	23	83	54	3.9	2.66–5.8	< 0.0001
Age ≥ 70	68	16	59	38	3.2	2.08–4.79	< 0.0001
Male gender	178	43	91	59	1.9	1.32–2.79	0.0007
Obesity and hypertension	364	88	44	29	2.7	1.72–4.28	< 0.0001
Diabetes and hypertension	83	20	68	44	3.5	2.33–5.17	< 0.0001
Obesity and diabetes	43	10	41	27	3.1	1.94–5.04	< 0.0001
Obesity, hypertension and diabetes	32	8	36	23	3.6	2.17–6.12	< 0.0001

OR, odds ratio (unadjusted); CI, confidence interval.

## Discussion

This retrospective study revealed valuable information about the history, clinical features and disease course amongst patients who died of COVID-19 in a large district level hospital in Cape Town, South Africa during the first wave of SARS-CoV-2 infections.

What is striking in the comparison of the two burden of disease surveys between February 2020 and June 2020 is the similar amounts of admissions for routine internal medicine diagnoses but with an extra 205 COVID-19 cases during the month of June 2020. It seems the burden of internal medicine diagnoses (other than COVID-19) did not decrease or plateau during the peak of the first COVID-19 wave despite the so-called level five lockdown and theoretical hesitancy for patients to seek medical help in fear of being exposed to SARS-CoV-2 at healthcare facilities. Another finding is the notable increase in cases admitted for hyperglycaemic emergencies during June 2021 independent of COVID-19 cases. This may be explained by numerous hypotheses, including suboptimal treatment and monitoring of general medical conditions at primary care level because of being overwhelmed by COVID-19 cases and the possibility of suboptimal COVID-19 surveillance at the study hospital.

Coronavirus disease 2019 survivors were 12.3 (95% CI: 9.16–15.39) (*p* < 0.0001) years younger than those who died in this cohort. The OR of dying of COVID-19 seems to increase with age, peaking at age 60 years or older (OR: 5; 95% CI: 3.31–7.41; *p* < 0.0001), followed by a decrease in OR of dying amongst patients who have passed the age of 65 years (Table 3). Amongst patients who died of COVID-19 the prevalence of non-communicable diseases such as hypertension, diabetes mellitus and obesity were higher than those who survived COVID-19. Having any two or more of the above-mentioned comorbidities carried an OR ranging between 2.7 and 3.6 (Table 3).

The clinical profile and comorbidities present in our cohort of patients who died of COVID-19 reflected those reported by Boulle et al., male gender, increasing age, diabetes mellitus, hypertension and chronic kidney disease being major role players.^17^ The HIV seroprevalence at our study centre was 13%, which is lower than what was found in other local studies, including Boulle et al. (Western Cape; 16%) and Parker et al. (Tygerberg Hospital; 21%).^17,19^

The variability in history taking, note keeping and clinical impression of doctors treating patients with COVID-19 proved to be a challenge when recording variables such as comorbidities and complications from clinical records. We suspect that the prevalence of certain important comorbidities such as obesity, hypertension and smoking history may have been under-reported. Even though there was suspected under-reporting of acute complications in hospitalised patients, AKI seems to have been very prevalent amongst patients who died of COVID-19 and amongst the study population the presence of acute renal failure carried a high OR of dying from COVID-19 (Table 3).

When comparing the CXRs of those patients who died with those who survived COVID-19, there did not seem to be a significant difference in the extent of infiltrates. A remarkable number of patients died very shortly following admission. This can be because of various theories including severity of disease and timing of (late) presentation to hospital. Another factor possibly contributing to early death was the limited capacity for escalation of care offered at district level. Several patients demised whilst awaiting transfer to other facilities offering a higher level of care.

Admission criteria to the hospital’s four bed HCU were indirectly determined by the nearby TBH’s dedicated COVID-19 intensive care unit (ICU). Patients who required high flow nasal oxygen or intubation for severe COVID-19 were selected for admission to our HCU based on their age, comorbid conditions, functional status prior to getting ill and the absence of concurrent medical or surgical conditions requiring a higher level of care. Where possible, qualifying critically ill patients were transferred to TBH COVID-19 ICU for further critical care prior to needing intubation. Patient transfer to other facilities such as TBH COVID-19 ICU proved to be a significant challenge, with patients rapidly decompensating, limited ventilation capacity whilst awaiting transfer and significant delays in transfers being experienced daily because of overwhelmed emergency medical services just being some examples.

It is uncertain whether timeous transfer and escalation of care of patients with severe and critical COVID-19 may have altered clinical outcomes. Factors such as delay in escalation of care and transfer to ICU, whether because of logistical issues or lack of capacity, may have played a role in the observed short time from admission to death.

Upon the publication of the RECOVERY trial on 16 June 2020 the use of systemic glucocorticoid therapy were included in national clinical guidelines.^31^ The addition of glucocorticoid therapy was followed by a reduction in the case fatality ratio amongst patients admitted with COVID-19 from before (29.7%; *n* = 70) to after (25.3%; *n* = 84) 16 June 2020. Although small study numbers may limit definitive conclusions in terms of therapeutic benefit, plausible explanations for a decrease in mortality after the introduction of glucocorticoid therapy include direct benefit from treatment with systemic glucocorticoids and advances in therapeutic and intervention strategies as the pandemic progressed.

## Limitations

This was a retrospective study and data sets were not complete for all variables as they were dependent on the history taking, clinical impressions, diagnostic skills and note keeping of the clinicians who managed these patients. An example is patients being classified as overweight or not, as all patients did not have their weight and height measured on admission.

We suspect that the cause for the disparity in the data were due to the under-reporting of acute complications and comorbid diseases. Even though all possible care has been taken to identify all cases admitted to the study centre with COVID-19, the possibility exists that the total number of COVID-19 cases is under-reported because of lack of a robust system where discharge diagnoses are recorded.

## Conclusion

This study describes the experiences of a district level hospital during the first wave of the COVID-19 pandemic. Given the small study population, incomplete data sets and numerous variables, limited definitive conclusions can be drawn from this study.

Patients who died of COVID-19 seemed to have similar comorbidities that are associated with increased risk of death. Amongst the study population, AKI seemed to have been very prevalent in patients who died of COVID-19. As a result of challenges with accurate and consistent note keeping and lack of standardisation leading to inter-clinician variability of clinical impressions, some comorbidities and complications seemed under-reported.

Apart from late presentation to hospital, limited ICU beds and logistical reasons causing delays in escalation of care contributed to a significant number of patients dying of COVID-19 very shortly after admission to the study hospital.
